# Milk-Sickness and the Illinois Legislature

**Published:** 1867

**Authors:** W. Anderson

**Affiliations:** Pontiac, Ill.


*Milk-Sickness and the Illinois Legislature.* By W. ANDERSON, M.D., of Pontiac, Ill.

[As the Illinois Legislature has adjourned without any action upon this matter, unless, perchance, the record thereof is among the contents of some yet unloaded "omnibus"—the fears of our correspondent are probably groundless. We insert his paper, however, for its bold challenge of a popular and *quasi* professional notion.—EDITOR.]

It appears from recent proceedings, that the Legislature of our State is about to reward a certain individual with a large donation for the supposed discovery of a new remedy for "*milk-sickness*:" the reward being subject to investigation and decision by a "*scientific committee.*"

This is an act of magnanimity that is very commendable, provided the said committee or Legislature is not grossly humbugged. Such acts of generosity have been passed by other Legislatures, and always, as far as I am informed, to their utter disgrace; and, from the facts in the case, there is but little likelihood that this will be an exception. The proposed recipient, Mr. B., may be a worthy gentleman, but no case of this kind could look more suspicious.

I think our Legislature would do well to first appoint a committee to ascertain whether there is such a disease as "milk-sickness," and if so, then, whether we have not already better remedies than Mr. B., or Dr. Brown, whatever he may be, is likely to bring forth. For if the affection has an individual existence at all, it is well known that it yields with equal readiness with bilious remittents to the same remedies, and it is my purpose to examine briefly whether they are not one and the same disease, originating from the same causes, and unconnected in any way with the milk of cows. I consider "milk-sick," as the vulgar call it, a mere matter of credulous fancy, and all I can see, hear, or learn in any way, on the subject, still further convinces me in this opinion; and my residence among it being yet brief, I think I can frame an opinion without bias.

A fact admitted by all, is, that the disorder is remittent in its character, and, in all particulars, resembles that form of fever, but they claim that it is caused by milk of cows that feed in timber along creeks and rivers.

Now, if the disease prevails only in miasmatic districts, resembles miasmatic fever, and is caused by cows that are subject to the same affection, that affection must be material, and could not arise from eating green vegetables, which is the universal pet theory here.

It is more reasonable to suppose the disease ordinary fever, which is always more aggravated along watercourses than on the prairies. Whether it is a reality or a myth is a question on which much light is needed to change my mind, and all I have yet learned is one-sided and negative. If I am wrong, I hope some physician of riper judgment, greater experience and wider views, will set me right.

In support of my views, I will not argue the question at length, but will only add the following considerations:—First, I have never known an irregular physician or empiric of any kind, but talked loudly of "milk-sick" and his ability to cure it, while genuine physicians generally ignore it entirely, or speak of it in terms of scepticism. Secondly, irregular works on medicine always treat of it as an individual and specific disorder, while professional works pass over it in silence, or allude to it cautiously. Thirdly, the existence of the malady only in excessively miasmatic districts. Fourthly, the more intelligent settlers in such district are more or less sceptical on the subject, while the credulous regard it as a heresy and a mark of incompetency in a physician to call their pet disease into question. Fifthly, most intelligent physicians who acknowledge its existence, confessedly do it for popularity and practice. I could continue such objections to great length if deemed necessary, but I purposed to be brief.

I presume there is not a class of men in the universe more ignorant of medicine and disease than politicians and legislative bodies; nor is there any other science in which mankind at large make so much pretension while they know so little, or are so easily bamboozled and humbugged. I repeat, the various Legislatures in this country have made similar appropriations before, but, as far as I am informed, they have been duped in every instance, while the hundreds of true cultivators of medical science have gone unrewarded. This is a shame and a slur on the human race.

It should not be forgotton that Pennsylvania voted many thousand dollars for a "*secret remedy*" for snake-bite, seneka snake root, namely, which is no remedy at all. And the New York Legislature likewise, fo ran antidote for hydrophobia, which is still utterly incurable.

So much for legislative prudence and wisdom on scientific subjects.

In conclusion, I will state, that my observation, as well as that of many able physicians and other citizens, is, that so-called milk-sickness is only the severer forms of miasmatic or bilious remittent fever, generally distinguished by the terms congestive, remittent, &c.; that it is rather fatal in its character, yields, if at all, to antiperiodics, cathartics, &c.; or, in other words, stimulants, during the congestive stage; quinia, between the paroxysms, in very large doses, and castor oil or croton oil as a physic, preceded or followed by calomel. Arterial sedatives will be of great benefit in case the fever runs high; but as to the new treatment of Mr. B. and the Legislature, I have not the remotest thought that there is anything in it.

